# Supplementary material for: Exploring perceptions of gender roles amongst sexually active adolescents in rural KwaZulu-Natal, South Africa
Source: PLoS One. 2024 Jan 18;19(1):e0296806. doi: 10.1371/journal.pone.0296806 (PMC10795977; doi:10.1371/journal.pone.0296806)
Supplement: S1 File — (PDF) [file pone.0296806.s001.pdf]

Session 1: Identity

Hello and thank you for agreeing to speak to me today. In today's session I would like to explore a few elements that influence what makes you who you are within the context of society. It's a few questions about relationships between men and women, culture and race. Do not hesitate to ask me to stop if you start feeling tired or uncomfortable.

I would like for you to take a little bit of time and think carefully about how would you describe yourself to someone who doesn't know you at all and cannot see or hear you?

- What makes you feel like you are a woman/man? (*Probes: Are there any rituals? Is it only because of your body?)*
- How do you think men and women are different? *(Probe: biologically/Psychologically/ Emotionally/ Socially)*
- Tell me what are some stereotypical ideas of what/how women and men 'should be'?
    - Are there some things you think women should not do?
    - Are there some things you think men should not do?
    - How do you think you are different from those?
- Are there some things you would like to do but cannot because you are a woman/man? *(Probe: manly vs womanly activities/jobs/behaviours-what are these things? Why do you feel this way?)*
- Do you think it's harder to be a woman or a man? *(Probe for contexts – at work? School? At home? Why is it harder/not harder in each of these contexts?)*
- Do you feel obligated to have children? Why? When *(age/relationship status/how many/fertility issues/gender of the child?)*

Cultural Identity

Now I would like to discuss customs and traditions and how they impact the way people think about men and women.

- What, do you feel, are your obligations as a Zulu woman/man? *(Probe: within your relationships/family/community)*
- Do you think that's different for people who are not Zulu? (*Probe: how do you feel about your culture in relation to other cultures?*)
- What are some clichés other South Africans have about your culture? Do you think they are true?

Racial Identity

Let's talk about your opinions on race, it is a complicated and important social construct, and it plays a key role in almost every aspect of South African society and I would like to know how you feel it impacts your life.

- What do you think about being a Black person in South Africa today*? (Probe: how is this different to what your parents may have experienced?)*
- Do you think it's easier to be [a] non-Black woman/man?
- Do you think it's easier to be a black woman or black man?

- What is the hardest part of being a Black woman/man?
- Do you have non-Black friends? Is it easy to have friends of different races? Interactions/socializing
- Would you ever date someone outside of your race? *(Probe: Why? Which race would you date first, why?)*
- Have you ever experienced moments where your blackness is a benefit? An impediment? *(Probe for details and situations)*
- In which situations do you feel most aware of your blackness? *(Probe: for instances of racism/sexism/colourism)*
- Do you think all Black people are treated equally in South Africa? *(Probe: colourism/xenophobia/tribalism)*

Thank you so much for taking the time to speak to me, I know this was long and detailed, so I really appreciate you taking the time. After you get home, if there's anything else you think about and that you would like to add, you can write it down in your diary or we can always talk about it at the beginning of our next session.

Session 2: Sexual Orientation and societal expectations

Hello, I hope you're feeling well. Thank you so much for coming back for our second session together. Last time we talked about your gender identity as well as the cultural and racial elements of your personality. Is there anything you would like to add to last time's conversation? Anything you thought about during our time apart?

Sexual orientation

Today, I would like to continue the conversation a little deeper. We are going to talk about sexual attraction. People can be attracted to men or women or both at different times of their lives or always. I know this can be a delicate conversation, please remember that you can choose to take a break or stop altogether at any time during our exchange.

- How do you define sexual orientation? Straight/Gay/MSM/lesbian/bi/queer/trans/non-binary/other *(Probe: how this may have changed over time? Does this change according to where you are/who you are with?)*
    - How would you define this in English vs isiZulu word adequate for your sexual orientation? Is there a word for your sexual orientation?
- What do you think about same-sex relationships? *(Probe: Individual perceptions vs community perceptions / why do you feel this way about same-sex relationships? / are there any repercussions for people in same-sex relationships where you live?)*
- Do you think it's ok to experiment sexually with someone of the same sex? *(Probe: what sort of same sex behaviour is acceptable?)*

- **LGBTQI**
    - Have you always known that you were gay/lesbian/bisexual? *(Probe: When did you realize you were gay? What age were you? Who did you tell? Why did you tell that person?)*
    - Does everybody around you know that you're gay? Who knows? How do you choose the people you tell? Has anyone ever outed you without your consent?
    - Do you feel accepted as a gay person? Are there spaces where it's harder for you to be gay? *(Probe: which spaces? Why?)*
    - What do people expect gay people to be like? *(Probe: Flamboyant, feminine, masculine etc.)*
    - Have you ever had to pretend not to be gay? When? Why? (how do you pretend not to be gay?)
    - How do you resolve your sexual identity with your day-to-day identity?
    - Both Blackness and queerness can present some challenges, do you think it's harder to be Black or to be gay? Why?
    - Do you think it's easier for non-Black people to be gay? Why?
    - Is there a space where you feel comfortable being yourself, both Black and gay?
    - How are gay Black people represented in the media? *(Probe: Do you think it's accurate? Stereotypical? Diverse?)*
    - Do you have a role model that you identify with? Who? Why?

Religion and Society

In the final section of today's chat, I would like to talk about your spiritual journey and how it impacts you.

- Would you define yourself as a religious person? (*Why? Why not?*)
- How did you choose your religion?
- If you are religious, what is the purpose of religion in your life? *(Probe: impact on morality, principles, behaviour and decision making?)*
- If you are not religious, why aren't you? Do you think it's acceptable to those around you (family)? Are you open about the fact that you are not religious?
- If you are religious, what do you think about people who are not religious?
  - **HOMOSEXUAL/BISEXUAL**: If you are religious and gay/bisexual, how does your religion impact how you feel about your sexuality?
- If you are religious, are you comfortable with the way your religion treats gay people? (not sure if it's a separate probe as it might already be answered in this question, but it would be interesting to see how people who are both gay and religious navigate this)
- If you are religious, are you comfortable with the place of women in your religion or in your church? *(Probe: Is there a difference in the way men and women are treated? If yes, would you like to see that change and how?)*
- If you are religious, do you ever seek guidance about your sex life in church/from your pastor/religious leader?

Thank you so much for taking the time to speak to me, I know we spoke about some very personal and intimate things, so I really appreciate you taking the time. After you get home, if there's anything else you think about and that you would like to add, you can write it down in your diary or we can always talk about it at the beginning of our next session.

Session 3: Sexual Behaviours

Hi there, it is so great that you are back to talk with me again today. How have you been?

- ***How is your family doing? How about your friends, have you guys been doing anything interesting that you can tell me about?***
- ***I hope that you enjoyed the last discussion we had about your identity and how your experiences as a young women or man or as straight or gay person, have influenced who you are. I feel privileged to have you share your experiences with me.***
- ***Is there anything you want to talk about regarding the last session we had?***

Today we are going to talk about your sexual development, remember that you should feel free to discuss your experiences with me, I will not judge you, and I will keep what we discuss confidential. However, if you do not feel comfortable sharing some information, you should feel free to say so, and we will move on to another question.

Ok, let us get started. Let us talk about your thoughts on young people and their sexuality.

Opening questions

- What do you think of as sexuality? (How would you define sexuality?)
- How comfortable is your family with speaking about issues related to sexual health? *(Probe: have you discussed issues like sex, contraception, pregnancy, STIs/ who do you speak to the most about these issues)*
- What have you learnt about sex and relationships from your family, either directly or indirectly (by observing them)?
- Have you spoken to your family about puberty? Changes you went/going through that have changed how your body looks? *(Probe: why/why not, who they spoke to, why they chose that person).*
- Have you spoken to your friends about puberty? Changes you went/going through that have changed how your body looks? *(Probe: why/why not, who they spoke to, why they chose that person).*
- Who do you speak to about the boys/girls you like? *(Probe: what do you talk about?/ why that person).*
- What do you do think is important for having a good relationship?

Abstinence

There are many types of things people do when they are exploring their sexuality. These could be things that they do alone or with someone else.

All of us start out not having sex, or maybe thinking about sex or sexual behaviour but not doing anything. People often say that we are practicing abstinence.

- What do you think being abstinent means?

- Tell me about your experience of being abstinent? *(Probe: how did it make you feel? Did you still have sexually thoughts? What did you do when you had these thoughts? How long were you abstinent for?)*
- When did you feel ready to start exploring your sexuality? This may mean that you had not done anything sexually (alone or with someone else) yet (what made you feel you were ready too? How did you make this decision? Who influenced this decision? How old were you?) (Probe around solo activities vs partnered activities?)

Early Solo Exploration:

Many people explore their sexuality alone first; this may be by watching or looking at porn, masturbating alone, or even masturbating with other people.

- Can you tell us about your early sexual exploration? *(Probe: Why did you start doing this, how did you feel afterwards, did you feel pressure to engage in these activities, did you speak to anyone about it?)*
- Tell us about why you think people masturbate?
- Did you masturbate before you started having sex with others? *(Probe: What age did you start? Why/why not/ how do you feel about doing this? Is it ok for boy's vs girls to do this? How frequently do you masturbate? Does it help you know what you enjoy and does it inform how you then have sex? Did you ever masturbate with other people?)*
- Did you date anybody before you started having sex? Tell me about people you dated but didn't have sex with (probe for details of partner/length they dated for).
- Many young people are now using dating apps, or online apps etc to talk to/date people without necessarily then having sex with them.
  - *Have you ever had online encounters with someone but you did not necessarily plan on having sex with them? (When was this/ did it involve sexting/sharing pictures (explicit or non-explicit/grindr/tindr other sites for hook-ups/videos/video calling)?*
- How did you start to learn about sex? What did you think was the "right way" of having sex?
- What do you think is proper sex?
- Many young people learn about sex, or find pleasure from watching porn? What do you think about watching porn?
  - *Why do you think people watch porn?*
  - *Where do you access porn from? (Where do other access it from?)*
  - *Do you think it teaches you about how sex should happen?*
  - *Do you think boys and girls watch porn?*
  - *Do you think it is good or bad?*
  - *Do you ever feel ashamed about watching porn? / do you ever watch porn and have sexual fantasies that make you feel ashamed? (all porn vs certain porn? Why?)*
  - *What do other people think about watching porn?*
  - *Do you think its ok to watch porn when you are single? Do you think it is ok to watch porn when you are in a relationship?*

    - *Have you watch porn while being in a relationship? (Probe: which relationship, why, how did you feel about it?)*
    - *How do you think porn is the same as real sex? How do you think it is different?*
- What do you think it means to be sexually active?
- When do you think a person has lost their *virginity*?

Session 4: Sexual debut and partnered sexual experience (and continuation of solo sexual acts)

We are going to start talking about when you started to have sex with a partner/hook-up/casual partner. Remember that you can let me know if you do not want to answer a question and we will skip to another question.

- Why do people your age have sex? *(Probe about Status, pleasure, intimacy, love i.e., the symbolism of sex. Also, Peer group influences, the courtship process)*
- How do you know if somebody wants to have sex with you? *(Probe about If you want to have sex with somebody what do you have to do to show them? If you want to have sex with somebody and they do not reciprocate what do you do? If you do not want to have sex with somebody how do you tell them? What cues would you take as a person wanting to have sex?)*
- Before having sex for the first time had you done anything previously. *(Probe: i.e., making out, oral sex etc. – light sexual activities, details of the partner [age, sex])*
- How do people your age generally react if they find out somebody has engaged in 'light' sexual activities versus actual sex? (*Probe: Is it different for girls vs boys/what would people think of as light activities/does it impact on your reputation amongst friends/ does it impact your reputation amongst the opposite sex and family/ is it more acceptable to engage in light activities vs full sex?*)

Sexual Debut

- Would you be able to tell me about your first sexual experience? (Probe: *age, sex of partner, partner age, what they did, did them speak about what they were going to do? was there foreplay? Did you think you were ready to have sex when you did? How did you feel afterwards? Who did you speak to about it? How did they react when you told them? Was it what you expected it to be? How did you protect yourself from getting pregnant/making someone pregnant and HIV/STIs?)*
- What motivated you to have start having sex? *(Probe: status, pleasure, intimacy, love, peer group influences, natural progression of the courtship process)*
- Did you feel you were ready for sex? / How does someone know when they are ready to have sex? *(Probe: Are there certain milestones they must hit first? What are these milestones? Is it obligatory once someone hits a certain age? Or a certain length of relationship? Forced/pressure to have sex/ partner expectation/boredom)*
- How did you decide that you ***were both*** *ready* to have sex? *(Probe: mutual negotiation, spontaneity, the man decided etc. – Also probe on the setting, where did it first take place)*
- What were your expectation(s) about the first time you had sex? *(Probe: Was it the same as what you have learned about before having sex with someone? Has this changed after sexual debut? Was it enjoyable?)*
- Did you first have sex with a long-term / short-term partner/casual hook-up/friend? Older or younger? *(Probe as to why they chose this person)*
- After you first had sex, did you start having sex more frequently? (*Probe: Did it feel easier to then continue having sex/ did sex become more steady and regular, less regular or did you stop – probe why this was the case)*

Sexual History post Sexual debut

We would like to talk to you about your sexual history after you had sex for the first time

- Have you continued to have sex, or engage in other types of sexual behaviours? *(Probe for secondary abstinence, types of sexual activities engaged in)*
- How important is sex for having a good relationship?
- How many people have you ever engaged in some sort of sexual behaviour with? *(probe for penetrative sex (vaginal or anal), oral sex? Masturbation, Mutual masturbation? Other [probe for age, sex of these partners])*
- Have you ever had a once-off sexual encounter (a hook-up)? How did this happen? What made you decide to do this? Did you think about HIV or pregnancy issues? How did you feel afterwards?
- How often do you engage in foreplay? *(probe – understanding of foreplay, do they like it, how is it constructed)*
- **Heterosexual participants:** Many young people experiment with their sexuality during adolescence, but they may feel ashamed to talk about it. Often this includes experimenting with people who are the same sex as them
    - Have you ever engaged in some sort of sexual activity with someone who is the same sex as you? (What sort of activity was this, how did you feel afterward? Did you ever feel comfortable to talk to someone about it?)
- **Homo/bi sexual participants:** Many young people experiment with their sexuality during adolescence. Have you engaged in some sort of sexual activity with people who the opposite sex to you?
    - Have you ever engaged in some sort of sexual activity with someone who is the opposite sex to you? (What sort of activity was this, how did you feel afterward?)
- What are your personal beliefs about multiple partners? (Probe: *Is it wrong to have multiple partners? Is there a problem with having multiple partners if you take precautions to prevent negative outcomes like HIV, STIs, pregnancy etc.?)*
    - Have you ever have/had more than one person you are engaging in some sort of sexual or ***relational behaviour*** with, this could be sex/flirting/sharing explicit pics etc.? *(Probe why do you have these multiple relationships/ why do people do it? How do you describe these partners (causal/long-term)/have you just ever hooked-up once off?)*
    - When is it okay for a girl/guy to have sex outside of a relationship?
- Do you think condoms are necessary to use in all sexual encounters? *(Why?) What type of sex behaviours do you think need a condom? (Penetrative sex, oral sex, mutual masturbation etc). When is it no longer important to use condoms? When can stop using them in relationships? Have you used them consistently in your previous relationships/sexual encounter? What is good about condoms, what is bad about them? Do you like them?*

Session 5: Current sexual Partner/s and general beliefs

- Would you be able to tell me about your current sexual experiences? (Probe: *number of current partners, age, partner age, what they did, did they speak about what they were going to do? was there foreplay? Did you think you were ready to have sex when you did? How did you feel afterwards? Who did you speak to about it? How did they react when you told them? Was it what you expected it to be? Types of behaviours you have done with your partner/s – oral, masturbation etc.)*
- *Do you think it is important for your partner to orgasm during sex? How do you know that they have? Is it important for you to orgasm during sex?*
- *Do you guys communicate about sex? (Probe: talk about the things you like/what went wrong/what you did not like/what they did not like?)*
- What motivated you to have start having sex with your current partner/s? *(Probe: status, pleasure, intimacy, love i.e., the symbolism of sex. Also, Peer group influences, the courtship process, pressure, relationship length, boredom)*
- Do you feel you have better control/power over sex now? *(Probe: power in relationship/protection behaviours- condoms/PrEP/contraception other).*
- How often do you engage in foreplay? *(probe – understanding of foreplay, do they like it, would they tell their partner, how is it constructed)*
- Do you currently engage in some sort of sexual or *relational behaviour* with other partners, this could be sex/flirting/sharing explicit pics etc.? *(Probe why do you have these multiple relationships/ why do people do it? How do you describe these partners (causal/long-term)/have you just ever hooked-up once off?)*
- Have you ever had sex even if you did not feel like it? (Probe: Why? Which partners? What did you do about it? Do you feel comfortable saying no when you don't want to have sex? What happens when you say no?)
- How do think you are dependent on your partner? (Probe: Emotionally, financially etc.)
- How do you manage your sexual health now? (*Probe for protection against HIV/STI/pregnancy etc condom use [when did you stop using it/how did you decided this/was this your decision or your partners?]*).
- Do you masturbate even though you are having sex? (*Probe: why/why not/ how do you feel about doing this? Is it ok for boy's vs girls to do this? Does it help you know what you enjoy and does it inform how you then have sex?)*
- Do you prefer masturbation or sexual intercourse (Probe: Why? Pleasure? Convenience? Control? What is different between the two?)
- Have you ever been asked by a partner, or asked a partner to engage in other sexual activities? I.e. group sex/threesomes/ same-sex activities/voyeurism activities etc. *(Probe: how did you feel about that? Are there any activities that you / your partner believe are immoral / are off-limits? Why?)*

Sexuality and other sexual behaviours and pleasure

- Do you enjoy sex, what about it is enjoyable? (what makes it enjoyable? What makes it good?)
    - How do you know your partner is turned on? How do you know if you are turned on?

    - Is your pleasure or your partner's pleasure more important to you? (Probe: why?)
    - How do you increase your pleasure? And your partners pleasure when you have sex?
    - How do you know if a person you are having sex with, or with who you are engaging in any sort of sexual activity has enjoyed it?
    - Is pleasure important for sex? *(Probe: is this the same for both people involved? Is one person more important than the other?)*
- What would your ideal sexual experience be? *(Probe: how should it happen?)*
- How do you make decisions about when you have sex?
    - When do you include the feelings of person you are going to have sex with in this decision? *(Probe: Does this depend on the partner? i.e. is it different for a longer term relationship vs a hook up?)*
    - Do you think your decision to have sex, and what you are willing to do depends on how horny you are? *(Probe: How is it different? Do you have any regrets afterward?)*
- Same sex activities:
    - **ALL:** What sort of same sex experimentation do you think people would find acceptable?
    - **GAY/BISEXUAL MEN:** Is it more acceptable to be a top or a bottom or versatile?
    - **LESBIAN OR BISEXUAL WOMEN:** Is it more acceptable to be a feminine or masculine lesbian?
    - When your heterosexual peers are talking about sex how do you feel / what do you talk about?
    - Is the courtship process different to the conventional heterosexual courtship process?
    - Where do you have sex? (setting)
    - What risks are present (this could be sexual risks as in STI's and also systemic and discriminatory risks, how is this negotiated)

Session 6: Forced sex/Gender/community beliefs about sex

- What age would you advise younger girls/boys to have sex at? How is that different to the age you first had sex? (Probe: Why?)
- What is the typical male role in sexual relationships / what is the typical female role in sexual relationships? *(Probe: Women – when are they allowed to initiate sex/ who has control/can women and men act on their urges equally/Men – How would you react if she had condoms with her and insisted you use them?)*
- Do you feel like you must be good at sex? How does somebody become good at sex? What makes somebody good at sex*? (Probe: different for males and females? Communication about if it is good or not? How do you know if it was good? How do you assess if it was good for you vs your partner?)*
- How often have you gotten ideas about things to do sexually from talking to your friends? / Where do you get your sexual ideas from typically?
- How often have you gotten ideas about things to do sexually from asking your partner what kinds of things they like?
- Have you ever had sex even if you did not feel like it? *(Probe: Why? Which partners? What did you do about it? Do you feel comfortable saying no when you don't want to have sex? What happens when you say no?)*
- Have you ever chosen a partner because he will take care of you? How did he take care of you? What did you give him in return? *(Probe: Age of partner, how long relationship was? Did you feel happy about it? Did you feel pressured to stay in the relationship?)*
- Have you ever had sex in exchange for something you didn't have? *(Probe: why? What did you get? How did you feel about it?)*
- Have you ever exchanged explicit pictures / videos / engaged in sexting for something that you didn't have? (Probe: why? What did you get? How did you feel about it?)
- Have you ever been forced to have sex or do something you were not comfortable doing? *(Probe: Can you tell me about what happened/ how did you deal with it)*
- Do you know / have you ever heard about lesbian women being forced to have sex with men that they did not want to have sex with? (probe: Why does this happen?)

Closing question

- How do people in your community feel about sex amongst young people? *(Probe: older people, parents, peers [male vs female], how would they feel about people having sex at different ages [15 vs 19], when is acceptable in the community for people to have sex [girls vs boys]?)*

<u>Photovoice</u>

- LGBTQ: Private intimacy vs 'communal' intimacy
