# Supplementary material for: Exploring perceptions of gender roles amongst sexually active adolescents in rural KwaZulu-Natal, South Africa
Source: PLoS One. 2024 Jan 18;19(1):e0296806. doi: 10.1371/journal.pone.0296806 (PMC10795977; doi:10.1371/journal.pone.0296806)
Supplement: Attachment — Submitted filename: Reviewer Report.docx [file pone.0296806.s002.docx]

Reviewer’s report

Overview

The manuscript addresses an important topic on gender roles vis a vis adolescents’ sexual reproductive health among adolescents in South Africa. Indeed, the study “highlights “how gender roles and social expectations may influence sexual decision making and how the social push for bearing children may be in conflict with messaging around the possible negative health outcomes of adolescent pregnancy, and implications for sexual reproductive health interventions.

The manuscript is well written and the sections flow well. The manuscripts will be strengthened by addressing the following minor comments.

Minor comments

Methods:

Participants’ demographics

1. It would be helpful if the authors could include demographic information (at school or of out of school, employment if any). This would help shed light regarding the findings as well as illustrate the dissonance between perceived gender roles and reality e.g. if any of the participants especially women had jobs to help make ends meet in their families.

2. Lines 109-110, (page 5). “The age range was included because it included those mid-adolescence therefore reducing potential recall bias about early sexual behaviour” and justification for the selection in lines 110-113 is not clear, please simplify or revise.

Discussion

The discussion section is well articulated. As noted in the study: “Men and women were perceived as diametrically opposed which stimulated the idea that men and women should fulfil differing, and often opposing, gender roles.” The results seems to suggest that some of the views, although minor indicate a ‘shift’ from culturally held views e.g. male adolescents with interest in activities such as cooking (considered female roles); and the anomaly re: women’s role being household heads yet perceived as subservient to men. It would be helpful to reflect on this minor theme/s and the implications going forward.

Lines 347-348 please adjust alignment/justification of the text.
