# Supplementary material for: Exploring perceptions of gender roles amongst sexually active adolescents in rural KwaZulu-Natal, South Africa
Source: PLoS One. 2024 Jan 18;19(1):e0296806. doi: 10.1371/journal.pone.0296806 (PMC10795977; doi:10.1371/journal.pone.0296806)
Supplement: Attachment — Submitted filename: Response to Reviewers_09 August 2023.docx [file pone.0296806.s003.docx]

Dear Obasanjo Afolabi Bolarinwa,

Thank you for sending through the comments from the reviewers. We are appreciative of the time and effort put in by the reviewers and have responded to the comments. These are provided in the table below.

In addition, two responses to queries from the journal are written below as requested:

- Funding Statement Should read: Financial Disclosure Statement. This work was supported by the South Africa National Research Foundation’s Thuthuka Funding under Grant [number TTK200403511178]. There was no additional external funding received for this study. The funders had no role in study design, data collection and analysis, decision to publish, or preparation of the manuscript.
- Data Availability Statement Should read: All relevant data are within the paper and its Supporting Information files. We have included the questionnaire guides within the supporting documentation.

Kind Regards

Hilton

**Tabulated Response to the Reviewers**

| Comment | Comment | Edit |
| --- | --- | --- |
| Journal Requirements Naming style | Please ensure that your manuscript meets PLOS ONE's style requirements, including those for file naming. The PLOS ONE style templates can be found at  https://journals.plos.org/plosone/s/file?id=wjVg/PLOSOne_formatting_sample_main_body.pdf and  https://journals.plos.org/plosone/s/file?id=ba62/PLOSOne_formatting_sample_title_authors_affiliations.pdf | The document has been checked and conforms to the journal style guide. |
| Journal Requirements  Points 2 & 3 | Thank you for stating in your Funding Statement: "This work was supported by the South Africa National Research Foundation’s Thuthuka Funding under Grant [number TTK200403511178]."Please provide an amended statement that declares *all* the funding or sources of support (whether external or internal to your organization) received during this study, as detailed online in our guide for authors at http://journals.plos.org/plosone/s/submit-now. Please also include the statement “There was no additional external funding received for this study.” in your updated Funding Statement. Please include your amended Funding Statement within your cover letter. We will change the online submission form on your behalf. | Thank you so much for bringing this point to our attention, we have made the required changes: Financial Disclosure Statement. This work was supported by the South Africa National Research Foundation’s Thuthuka Funding under Grant [number TTK200403511178]. There was no additional external funding received for this study. The funders had no role in study design, data collection and analysis, decision to publish, or preparation of the manuscript*.* |
| Journal Requirements 4 – Data Availability Statement | Thank you for stating the following financial disclosure:  "This work was supported by the South Africa National Research Foundation’s Thuthuka Funding under Grant [number TTK200403511178]."Please state what role the funders took in the study. If the funders had no role, please state: ""The funders had no role in study design, data collection and analysis, decision to publish, or preparation of the manuscript."" If this statement is not correct you must amend it as needed. Please include this amended Role of Funder statement in your cover letter; we will change the online submission form on your behalf. | Thank you for directing our attention to this issue, it has been updated in the manuscript, as seen in the comment above. |
| Data Availability | 4. In your Data Availability statement, you have not specified where the minimal data set underlying the results described in your manuscript can be found. PLOS defines a study's minimal data set as the underlying data used to reach the conclusions drawn in the manuscript and any additional data required to replicate the reported study findings in their entirety. All PLOS journals require that the minimal data set be made fully available. For more information about our data policy, please see http://journals.plos.org/plosone/s/data-availability.  Upon re-submitting your revised manuscript, please upload your study’s minimal underlying data set as either Supporting Information files or to a stable, public repository and include the relevant URLs, DOIs, or accession numbers within your revised cover letter. For a list of acceptable repositories, please see http://journals.plos.org/plosone/s/data-availability#loc-recommended-repositories. Any potentially identifying patient information must be fully anonymized. | Thank you for this comment. The statement should read: All relevant data are within the paper and its Supporting Information files. We have included the questionnaire guides within the supporting documentation. |
| Reviewer 1 (Methods) | It would be helpful if the authors could include demographic information (at school or of out of school, employment if any). This would help shed light regarding the findings as well as illustrate the dissonance between perceived gender roles and reality e.g., if any of the participants especially women had jobs to help make ends meet in their families. | Thank you for the comment. We have inserted a descriptive table for the demographic data of the cohort (see Table 1). |
| Reviewer 1 | Lines 109-110, (page 5). “The age range was included because it included those mid-adolescence therefore reducing potential recall bias about early sexual behaviour” and justification for the selection in lines 110-113 is not clear, please simplify or revise. | Thank you for the comment, we have edited the document to clarify these issues, “This age range was selected because it included those in mid-adolescence and are likely have had their sexual debut more recently, reducing potential recall bias about early sexual behaviour. This age group is also likely to be in similar sorts of sexual relationships (i.e., short term) compared to older adolescent groups where longer-term and formal relationships are more likely to occur and drivers of sexual behaviour may change.” |
| Reviewer 2 | The discussion section is well articulated. As noted in the study: “Men and women were perceived as diametrically opposed which stimulated the idea that men and women should fulfil differing, and often opposing, gender roles.” The results seem to suggest that some of the views, although minor indicate a ‘shift’ from culturally held views e.g. male adolescents with interest in activities such as cooking (considered female roles); and the anomaly re: women’s role being household heads yet perceived as subservient to men. It would be helpful to reflect on this minor theme/s and the implications going forward. | Thank you for the comment, we agree highlighting this nuance is important. We have added the following text: “Some results suggest that there may be some ‘shifts’ from culturally held views. Some male adolescents showed an interest in activities such as cooking (considered female roles). This may suggest that young men are open to expanding their definitions of the male role and highlights the need for interventions amongst younger men, changing cultural views over time as these men get older. Additionally, we saw an anomaly with regards to a women’s role as household providers. Even if they played a provider role they are still perceived as subservient to men, suggesting that economic empowerment interventions aimed at women may not be sufficient to led to shifts in gender roles, and norm and that gender equality interventions should be part of all social and health interventions.” |
| Reviewer 2  (Introduction) | Paragraph 2 appears to link adverse sexual outcomes with young women’s behaviours and decisions. For example, the sentence in Lines 61-64 appears to attribute experiences of violence to women’s ability to make decisions (when, by definition, acts of violence are taken by force). And the terms ‘risk-taking’ and ‘risky sexual behaviours’ imply individual agency or choice, with an implication of irresponsibility. The relationships (conceptual) between social norms, individual agency, and health and violence risks could be made clearer.  It would be helpful to distinguish social and gender norms, as they seem to be used interchangeably. Please also define ‘gender success’ and was it defined from young people’s perspectives in this research?  Also, the terms “positive” and “negative” are used frequently to describe gender and social norms but have not been defined. Which values or whose values are used to make these descriptions? | Thank you for this comment. We agree the wording may have implied more agency than people can exercise in many situations. We have reworded this to be clearer: Gender inequalities, unequal power dynamics, socially sanctioned gender roles and harmful gender and adolescent sexuality norms are important mediators of increased vulnerability to negative SRH outcomes amongst AGYW(1,5). Studies looking at gender norms (social norms defining acceptable and appropriate actions for women and men in a given group or society) indicate that unequal relational power between men and women, coupled with traditional beliefs about masculine and feminine gender norms, enhance risk of negative SRH outcomes (6–10). Unequal power dynamics and harmful gender norms also have a negative impact on women’s ability to make independent SRH decisions, increasing their risk of sexual and gender-based or intimate partner violence, and promotes negative gendered expectations (i.e., girl children dropping out of school to provide support at home) that keep women dependent on their partners/families and perpetuate gender inequality (1,11,12).  We have added a definition of gender norms as outlined in Chetty-Makkan, C.M., Grund, J.M., Muchiri, E. et al. High risk sexual behaviours associated with traditional beliefs about gender roles among men interested in medical male circumcision in South Africa. AIDS Res Ther 18, 33 (2021). <https://doi.org/10.1186/s12981-021-00359-7>.  We have made some edits which we hope will help clarify the notion of success as used in this paper. Bearing and fathering children was perceived as a key measure of successful womanhood and manhood by all participants. “Participants seem to rely on the ability to have children to gauge whether one has successfully grown into an adult, man or woman.”  We have clarified the issues around negative and positive gender norms to make this issue clear, the paper now makes it clear what we are referring to behaviours that promote gender norms that may have harmful implications. |
| Reviewer 2  (Methods) | There is little detail about the social context of the study setting, or why it was chosen. What are the schooling, marriage, ethnic and religion profiles in Vulindlela, to help interpret the findings as well as their applicability elsewhere? And to understand if the integrated theory of gender and power has validity in this socio-cultural context. | Thank you for bringing this to our attention, we have made additions to the section entitled Study Community From: Vulindlela is comprised of a population of just under 160,000 predominantly isiZulu speaking people and is characterised by high community HIV prevalence (21), as well as high rates of STIs and teenage pregnancy (21,22).To: Vulindlela is comprised of a population of just under 160,000 predominantly isiZulu speaking people and is characterised by high community HIV prevalence (21), as well as low rates of marriages, high rates of STIs, high secondary school dropout rates and high teenage pregnancy rates (21,22). |
| Reviewer 2  (Study sample) | Study Sample: some aspects of the study sample are unclear, for example:  - The participants are sometimes described as 15-19 years (in lines 108 and 161) and other times aged 16-19. In lines 161-163, the age range is described as 15-19, yet neither age range for males nor females includes age 15.  - In line 110: it’s not clear what recall “bias” would be at play or how it would distort the results (differential recall among those with adverse sexual outcomes compared to those without such outcomes?)  - It seems more likely that there is selection bias (or limited representation), in that:  - (1) those willing to disclose their sexual activity (in screening questions) and participate were different to other adolescents unwilling to disclose this private information? Particularly if there are social expectations about teenage sexuality activity, and those expectations differ among males and females. (Is this why there were more male than female as participants? 19 vs 10)  - (2) the 29 participants may represent adolescents who become sexually active at an earlier age than others (by choice or not). What is the mean age of first sex in this context?  - Also, snowballing may have recruited young people in similar peer networks (extending the sampling bias) rather than expanding the diversity of adolescents.  - The Discussion states that you “sampled for representativity” – it wasn’t clear in the Methods how this was done, or what kind of representation was sought. | Thank you for addressing this discrepancy. It was an error; the initial anticipated age range was in fact 15 to 19 years old but no participant under the age of 16 was recruited. The overall age range was *16* – 19 years old, and the mean age of male participants was 17.5 years old (Age range 16-19 years old), and 17.7-year-old for female participants (Age range: 17 – 19 years old). This has been updated.  The issue of recall has been corrected for clarity. It reads: Purposive sampling was used to identify participants living in Vulindlela aged between 15-19 years old who self-reported being sexually active (having had sex in the last month). This age range was selected because it included those in mid-adolescence and are likely to have had their sexual debut more recently, reducing potential issues with the recall of early sexual behaviour. This age group is also likely to be in similar orts of sexual relationships (i.e., short term) compared to older adolescent groups where longer-term and formal relationships are more likely to occur and drivers of sexual behaviour may change.  We agree that there may have been selection bias and have added this to the limitations section of the paper.  The sample was selected to represent males and females across the age group inclusion criteria. We have clarified this in the paper and mentioned the limitations of representativity. |
| Reviewer 2  (Data and tools) | The data are described as “longitudinal”, with data from 2 interview rounds used for this analysis. Is the analysis treated as longitudinal, to identify changes with age or time? How was data analysed for those with only 1 interview, if lost to follow up, versus participants with 2 interviews?  Can the interview guides be included in supplementary material? Was the same guide used in both interviews?  Did the authors conduct a reflexivity statement to explore their own active role in the results generation? Can this be included in the submission material?  Related to this point, Braun & Clarke 2006 (the methodology cited) are critical of describing analysis in ways such as “additional codes emerged from the data” (line 150) since describing the analysis as a passive process denies the active role of the researchers.  Also, is ‘inter-rater reliability’ the goal in an analysis process that is inherently subjective?  How did the researchers determine when saturation was achieved?  Can the authors justify use of the integrated theory of gender and power, for analysis, rather than an inductive or grounded theory approach? | Thank you for the comment, it may have been unclear in the methods that this included data from only the first round of interviews of a longitudinal qualitative study. This is in the methods section, but we have made it clearer. Although we conducted several rounds of interviews, only the first one is included in this paper. This has been clarified in the methodology. All participants in the study completed the first interview.  Thank you for your request, the interview guides have been added to the supplementary material.  We did not do a formal reflexivity statement, but as experienced qualitative researchers, and as most of the study team included psychologists, we discussed our position in the research process, and discussed the themes and codes as a group, looked for negative/deviant cases and reports, and presented our findings to the community research support group to discuss findings. All this improves the process of the analysis. Interviewers also consulted their field notes. We have added some details to the section.  We appreciate the comment on inter-rater reliability and have changed this to refer to consensus building.  Saturation was determined when further observations and analysis revealed no new themes.  As we state in the analysis section, the theory provided a guide for the analysis. Being guided by theory is an accepted method for qualitative analysis and allows a structure for identifying important themes and concepts. The use of theory does not preclude the identification of themes outside the theoretical model/framework. We allowed for this process to occur as outlined in the analysis section. |
| Reviewer 2  (Results) | Can a coding tree be included with the submission, for more detail about the data and themes identified by the researchers? | Thank you for the comment. We have added a table highlighting the important themes identified during the analysis – these encompass the main codes the define the themes. |
|  | It was specified in Methods that both heterosexual and homosexual males were recruited, however, there is no distinction made by sexual orientation in the Results. Also, the findings seem to apply only to heterosexual relationships: is that the case? Should we conclude that gender norms and expectations (of heterosexual relations) are the same regardless of male sexual orientation, or was this not explored in the analysis? This seems relevant to an analysis of sexual relationship power, and an opportunity for learning from the diverse experiences of adolescents. | Thank you for your comment. The focus of this first paper was not on relationships but on perception of gender roles and both homosexual and heterosexual participants shared a similar understanding of what was expected of them by the definitions of gender roles in their community. While some of the male participants identified as homosexual but were not asked how their sexual orientation impacted their vision of gender roles and how they played out in homosexual relationships, if at all as issues of sexual orientation and role identity were discussed in later interviews and will be the focus of a future analysis. We thought it was quite interesting that gender roles were so similar across sexual orientations, and we have added note of this to the paper. |
|  | Could use of the theory of gender and power have led to findings that supported the theory, with its strong reliance on traditional gender roles and patriarchal influences? Thus impeding the analysis from finding more variation and complexity – a possibility highlighted in this relevant manuscript: https://www.ncbi.nlm.nih.gov/pmc/articles/PMC7170748/pdf/nihms-1540698.pdf  May it also have impeded learning from both heterosexual and homosexual males?  (Also, shouldn’t the Connell paper from 1987 be used as the primary reference for this theory?) | Thank you for this important comment. As we said, the theory provided a guiding framework, and provided a sound structure for exploring the data. However, we did allow for the identification of themes that may not have fit easily in the framework. This is highlighted by the discussion of the norms that go against the traditional practices i.e., men wanting to cook but not being allowed, women being providers but not getting automatic relational power. We anticipate that the interviews focused on sexual identity more nuance about how homosexual and heterosexual experiences of gender norms and social expectations differ. We have noted this in the paper as discussed previously. |
|  | It is difficult to interpret statements on ‘early’ pregnancy or childbearing without understanding what qualifies as early. From when (what age) is childbearing a social expectation for young women? And by what age would a young male like P0014 feel so bad if he has not demonstrated fertility? Also, are there social circumstances that are expected along with childbearing, e.g., the context of marriage or partnership, or economic stability? E18 suggests that expectations about early childbearing have changed in this community – why may social stigma about teen pregnancy (previously “a disgrace”) have changed? This also suggests it may not be embedded in tradition or social norms, if expectations of early pregnancy have recently increased. | Thank you for this comment. The study team followed WHO guidelines and used early and teenage pregnancy interchangeably. Social pressure placed on young women to have children may highlight a tension between parental and adolescent SRH concerns and perceived social expectations driving teenage pregnancy also referred to in this paper as early pregnancy. We agree that there seems to be an interesting relationship between the gender norm around fertility, which is why we discuss that while the norm my suggest teenage pregnancy is a problem, the push to prove fertility may inadvertently reward it - entering a strange relationship where the norm around early pregnancy is negative but is similar rewarded. We have tried to capture this in the paper.  There are limitations to our data, and unfortunately some of the details about age at which it becomes “bad” to not have proved fertility was not able to be determined. |
| Reviewer 2  (Discussion) | In the Discussion, links between gender roles and HIV / SRH outcomes are made, based on assumptions or other studies. Did the research explore these links more explicitly? E.g., how adolescents perceive and navigate tensions between pressures to prove fertility and prevent HIV/STI infection? (Again, it would help to see the Topic Guide to understand what was asked) | Thank you for bringing this to our attention. We have added the interview guide and the team agrees that the correlation between perceived obligation to prove one’s fertility and the need to remain HIV/STI uninfected, warrants further investigation. It is important to develop hypotheses about future areas of work that need to be explored and we have mentioned this in the manuscript. |
|  | As noted, early pregnancy, here and throughout, is presented as an adverse outcome but what is considered “early” pregnancy in this social context? (and in relation to the legal age of consent) And in terms of young women’s preferences and choice? | Thank you for pointing out the need to clarify the understanding behind the notion of “early pregnancy”. As detailed above, we followed WHO guidelines in defining the age of early pregnancy (pregnancy between the age of 15-19 years old).*.* |
|  | Lines 527-530 call for interventions to shift harmful gender norms among early adolescents, and the existing evidence base on numerous community-based trials, including SASA! and Stepping Stones and MTV Shuga, among other campaigns, which could have been cited for relevance and lessons. Also, comprehensive school-based health education (UNESCO-led curricula adopted by many countries) addresses this to an extent and could have been considered here too. Other research finds that power dynamics in heterosexual relationships are exacerbated by age disparities between females and older partners, but this isn't addressed in this analysis or in the interpretation. In such circumstances, focusing interventions on early adolescents or even older adolescents will have limited impact. (And programmes like Stepping Stones may have more impact, as they include adult men) | Thank you for highlighting these important programs. We have added reference to these programmes. We do believe that our recommendation stands firm, as intervention in young adolescents will lead to changes amongst younger people, who will grow up with those norms internalized. This will lead to long term change in behaviour and should be additional to any programmes focused on adolescents or adults. |
|  | Given the number (only 10 females) and ways that the participants were sampled, it overstates the findings to say that social norms (demonstrated by the participants) are “pervasive” or across different age groups. (line 472) For reasons listed above, there may be more diversity and variation that was not identified due to the way the sampling and analysis was conducted, and the choice of theoretical framework. | Thank you for this relevant observation. We have edited accordingly. Further, it suggests that the dominant definitions of gender and social norms align with more conservative definitions, and *seem prevalent* within the community, across different age ranges. |
